# Transcriptome analysis of the painted lady butterfly, *Vanessa cardui* during wing color pattern development

**DOI:** 10.1186/s12864-016-2586-5

**Published:** 2016-03-31

**Authors:** Heidi Connahs, Turk Rhen, Rebecca B. Simmons

**Affiliations:** Biology Department, University of North Dakota, Grand Forks, ND USA; Department of Biological Sciences, National University of Singapore, Singapore, Singapore

## Abstract

**Background:**

Butterfly wing color patterns are an important model system for understanding the evolution and development of morphological diversity and animal pigmentation. Wing color patterns develop from a complex network composed of highly conserved patterning genes and pigmentation pathways. Patterning genes are involved in regulating pigment synthesis however the temporal expression dynamics of these interacting networks is poorly understood. Here, we employ next generation sequencing to examine expression patterns of the gene network underlying wing development in the nymphalid butterfly, *Vanessa cardui*.

**Results:**

We identified 9, 376 differentially expressed transcripts during wing color pattern development, including genes involved in patterning, pigmentation and gene regulation. Differential expression of these genes was highest at the pre-ommochrome stage compared to early pupal and late melanin stages. Overall, an increasing number of genes were down-regulated during the progression of wing development. We observed dynamic expression patterns of a large number of pigment genes from the ommochrome, melanin and also pteridine pathways, including contrasting patterns of expression for paralogs of the yellow gene family. Surprisingly, many patterning genes previously associated with butterfly pattern elements were not significantly up-regulated at any time during pupation, although many other transcription factors were differentially expressed. Several genes involved in Notch signaling were significantly up-regulated during the pre-ommochrome stage including *slow border cells, bunched* and *pebbles*; the function of these genes in the development of butterfly wings is currently unknown. Many genes involved in ecdysone signaling were also significantly up-regulated during early pupal and late melanin stages and exhibited opposing patterns of expression relative to the ecdysone receptor. Finally, a comparison across four butterfly transcriptomes revealed 28 transcripts common to all four species that have no known homologs in other metazoans.

**Conclusions:**

This study provides a comprehensive list of differentially expressed transcripts during wing development, revealing potential candidate genes that may be involved in regulating butterfly wing patterns. Some differentially expressed genes have no known homologs possibly representing genes unique to butterflies. Results from this study also indicate that development of nymphalid wing patterns may arise not only from melanin and ommochrome pigments but also the pteridine pigment pathway.

**Electronic supplementary material:**

The online version of this article (doi:10.1186/s12864-016-2586-5) contains supplementary material, which is available to authorized users.

## Background

Arguably, among the most striking examples of morphological variation are the stunning array of colors and patterns that decorate the wings of butterflies. The spectacular diversity of butterfly wing patterns has been shaped by natural selection to serve a variety of adaptive functions, ranging from mate recognition and courtship to predator avoidance and deterrence [1–3]. Although many of the ecological processes shaping color patterns are well documented, the underlying molecular and developmental program generating these patterns still remains largely unknown [1, 4, 5].

Over the past two decades, research has revealed that genes involved in wing color pattern development also belong to an ancient gene regulatory network (GRN) for wing construction [2, 6, 7]. This network has been proposed to serve as a pre-patterning template for downstream pigment genes [1, 8, 9]. Studies on wing development in *Drosophila melanogaster*, ants and aphids have characterized expression patterns of this gene regulatory network [10, 11]; however, no comprehensive analysis has been conducted in butterfly wings.

**Fig. 1:**
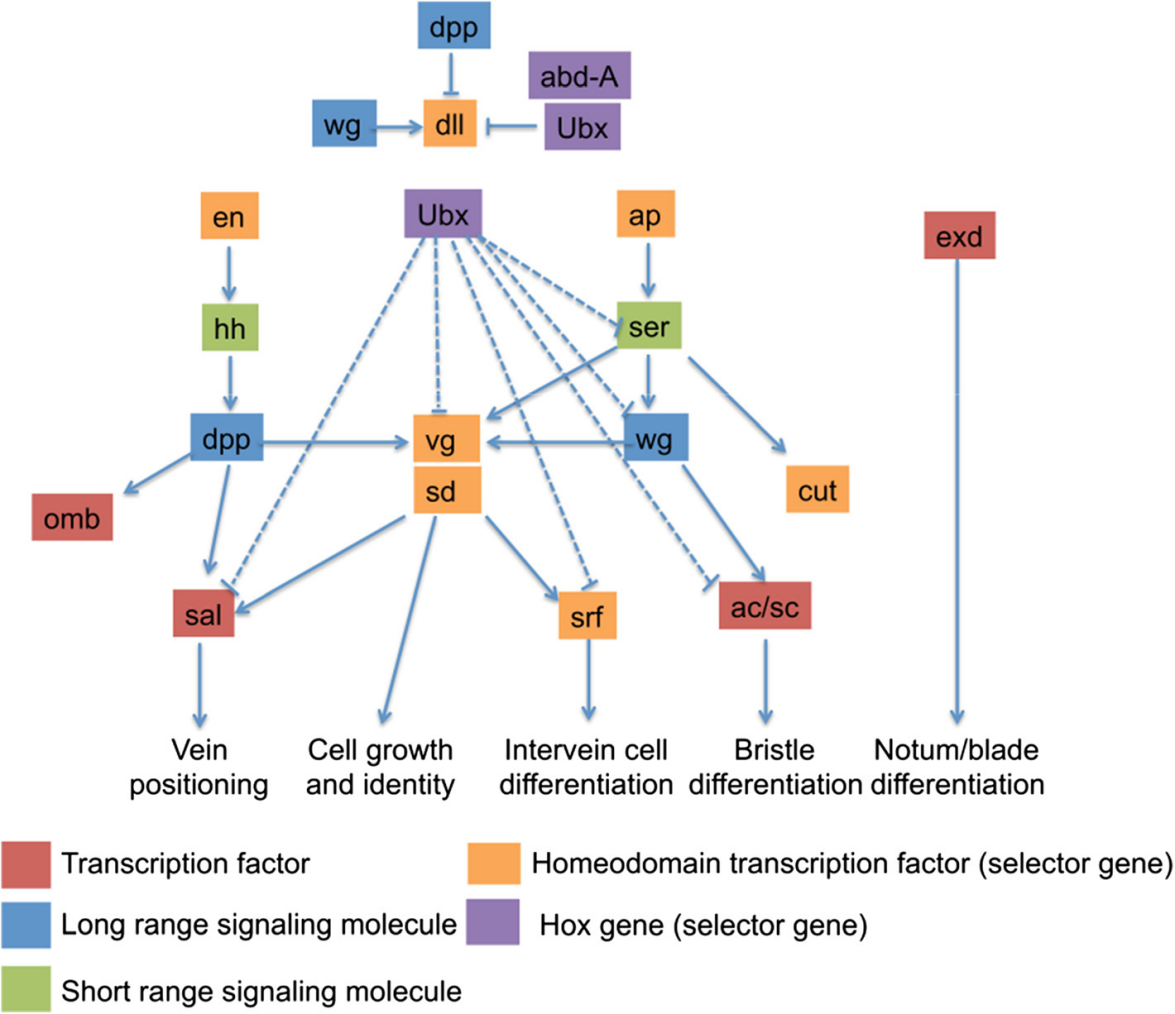
Wing gene regulatory network. Model of the gene regulatory network for wing development in *Drosophila melanogaster* adapted from [11]. The network depicts the hierarchy of patterning genes involved in the establishment of the imaginal disc and development of wings during the larval stages. Different functional groups are color-coded to highlight their role and placement within the network

The wing GRN characterized in *Drosophila* is comprised of least 18 developmental genes representing selector genes, morphogens and a suite of transcription factors that co-operate in wing development [11] (Fig. 1). Selector genes encode a unique class of transcription factors that act as master switches, controlling genes that regulate the development of specific cells, tissues and organs [12–14]. Selector genes include the Hox genes, which function as regional selector genes and specify segment identity along the anterior/posterior axis; one example is *ultrabithorax* (*ubx*) which regulates butterfly hindwing identity [15, 16]. At a finer scale, field-specific selector genes control growth of entire fields of cells and structures, whereas compartment specific selector genes regulate development of dorsal/ventral or anterior/posterior identity [13, 17].

In addition to regulating wing development, many of these selector genes and morphogens appear to have been redeployed in novel developmental contexts to specify wing color patterns, indicating a potential co-option event [1, 18–21]. Eyespots are the most well studied wing color pattern elements with at least 12 genes identified in the focus and colored rings [3, 19]. In nymphalid butterflies, expression of *antennepedia*, *en, sal, dll* and *notch* is observed in the focus of the eyespot [3, 19]*.* Many of these same wing developmental genes are also expressed in other pattern elements [18, 22]. These studies reveal a remarkably diverse role of these genes in controlling wing size and shape and also development of wing color patterns.

Wing color patterns are determined when each scale cell specifies a particular color pigment. A number of pigment pathways described in *Drosophila* have also been identified in butterflies including ommochromes (red, yellow and orange-- found only in nymphalids), and the melanins (black, brown and tan) [18, 23–25]. In general, ommochrome pigments appear earlier in pupal wing development than melanin pigments [26]. While many of the genes involved in pigmentation are well characterized, the connection between the developmental genes in the wing GRN and pigmentation pathways remains unclear [9, 27]. A link has been established between developmental genes and specific pigments; for example, *en* has been mapped to the ring of gold scales around the eyespots of *Bicylcus* [3, 19, 22]. Melanin pigmentation has also been shown to be associated with *sal* expression in pierid butterflies [28] and *wntA* signaling in *Heliconius* butterflies [4, 29, 30]. These examples implicate a role for patterning genes in regulating downstream pigment genes; however, identifying the gene networks and regulatory mechanisms linking the initial patterning process to final scale pigmentation remains an important challenge.

Next generation sequencing has become a valuable tool for surveying the transcriptome of non-model organisms [31]. Lepidoptera are a diverse order of insects, and there are still relatively few well annotated genomic resources [32]. Our current understanding of the genes involved in wing color pattern development is based on a small selection of species, primarily *Junonia coenia*, *Bicyclus anynana* and members of *Heliconius* [3, 27, 33–35]. A diversity of species should be examined to better understand how wing color patterning has evolved in butterflies. Here, we conduct a transcriptome analysis to examine the temporal dynamics of genes expressed during wing color pattern development in the nymphalid butterfly *Vanessa cardui*, with a specific focus on genes involved in patterning, pigmentation and gene regulation.

## Methods

### Tissue collection

*Vanessa cardui* caterpillars and artificial diet were purchased from Carolina Biological Supply Company (Burlington, NC). The caterpillars were reared individually at ambient temperature (~28 °C). Wing discs were dissected from caterpillars at two developmental time points in the final instar; early 4th larval (EL) and late 4th larval (LL) stages representing 2 and 4 days post-molt respectively, and at three time points during pupal development, early pupa (EP) 2 days, pre-ommochrome (PO) 5 days and late melanin (LM), 8 days post-pupation. Prior to harvest, larvae were weighed. The thorax, including the first abdominal segment, was harvested and placed immediately in RNAlater® (Ambion) and stored at 4 °C for at least 48 h prior to dissection. Pupal wings were dissected from live pupa using a Zeiss Stemi-2000 microscope and placed immediately in RNAlater and stored at 4 °C. Imaginal wing discs (fore and hind wings) were carefully dissected from the larva and placed in RNAzol® RT (Molecular Research Center Inc.) for RNA isolation. For pupal wing samples, fore and hind wings were placed in RNAzol for RNA isolation. All tissues were weighed and processed using an electric homogenizer followed by RNA isolation using isopropanol. Concentration of RNA was measured using a ND-1000 spectrophotometer (NanoDrop products, Wilmington, DE) (A260/A280 > 1.8) and integrity was assessed using electrophoresis on a formaldehyde-agarose gel. The RNA samples were diluted in water to a concentration of 25 ng/μl in 50 μl. All fore and hind wing discs were pooled for each larva prior to RNA extraction. RNA from 5 individual larvae was diluted and pooled for each developmental time point (in total four biological replicates of 5 pooled individuals per time point). A total of 11 larval libraries were prepared for RNA sequencing and transcriptome assembly. Two control libraries (one from early 4^th^ instar and one from late 4^th^ instar) were used for downstream expression analyses. The remaining libraries were part of a separate study involving treatment manipulations and were excluded from differential expression analyses. Following RNA isolation of pupal wings, forewings and hindwings were pooled for each individual and the RNA diluted as described above. For the 2 and 5-day pupal wings, diluted RNA from 4 individuals was pooled. Diluted RNA from 3 individuals was pooled for the 8-day time point. Two biological replicates of pooled samples were prepared for each pupal time point.

### Illumina sequencing and de-novo transcriptome assembly

Library construction was performed using the Illumina TruSeq RNA Sample Preparation Kit v2 (University of Utah Microarray and Genomic Analysis Core Facility). Briefly, total RNA (100 ng to 4 ug) was poly-A selected using poly-T oligo-attached magnetic beads. The Poly-A RNA was eluted from magnetic beads, fragmented and primed with random hexamers. First strand cDNA synthesis was performed using Superscript II Reverse Transcriptase (Invitrogen) and then converted to blunt-end fragments with an A-base following second strand synthesis. Adapters containing a T-base overhang were ligated to the A-tailed DNA fragments. The ligated fragments were PCR-amplified (12 cycles) and the amplified library purified by Agencourt AMPure XP beads (Beckman Coulter Genomics). Concentration of the amplified library was measured with a NanoDrop spectrophotometer. To determine the size distribution of the sequencing library an aliquot was resolved on an Agilent 2200 Tape Station. Quantitative PCR (KapaBiosystems Kapa Library Quant Kit) was used to calculate the molarity of adapter ligated library molecules and the concentration of the libraries was adjusted to a concentration of 10 nM. Library concentration was further adjusted in preparation for analysis on the Illumina HiSeq 2000 platform.

De-novo transcriptome assembly was performed using 50 bp raw reads in CLC Genomics Workbench (v. 6.5.1) with a word size of 40. The parameters were modified throughout the assembly and mapping process to optimize similarity (e.g. 0.96–0.98) and length fraction (e.g. 0.4–0.98). To control for assembly of chimeric sequences, contigs with read coverage less than 20 were selected at each step of the assembly for BLASTX searches against the nr database in NCBI. All sequences with less than 20 reads were discarded if BLASTX searches revealed potential chimeras. Mismatch, insertion and deletion costs were set at 2, 3 and 3 respectively.

### RNA-Seq analysis

CLC Genomics (v. 8.0) was used to identify differentially expressed transcripts by mapping reads from each library to the entire transcriptome assembly. The edgeR bioconductor package available in v. 8.0 was used to perform statistical analyses of read count data using TMM normalization [36]. Comparisons were made between LL vs. EP, (larval to early pupa) EP vs. PO (early pupa to pre-ommochrome) and PO vs. LM (pre-ommochrome to late melanin). The RNA-seq data were filtered to obtain transcripts ≥ 500 bp for reliable annotation and with a False Discovery Rate (FDR) *p*-value <0.001 for each comparison. This FDR cut-off allowed us to obtain highly significant results and a manageable number of sequences for annotation.

### Gene annotation

Sequence annotation was performed on the filtered sequences using a variety of approaches including local BLASTX (*E* value < 1 x10^-5^) to *Drosophila melanogaster* peptide database (FlyBase.org). Transcripts with no blast hits to *D. melanogaster* were annotated in BLAST2GO PRO by performing a BLASTX search against the entire non-redundant database at NCBI (*E* value < 1 x10^-5^). Following annotation, the transcripts were processed through the BLAST2GO pipeline [37].

To identify regulatory genes (transcription factors) and genes involved in pigmentation, gene names and symbols were obtained from the Animal Transcription Factor Database (bioinfo.life.hust.edu.cn/AnimalTFDB using *Drosophila melanogaster*) and Amigo2 (amigo2.geneontology.org) and matched to annotated contigs using header extractor (users-b0irc.au.dk/biopv/php/fabox) and the VLOOKUP function in Excel. Heatmaps were generated in JMP (v. 11.0) (SAS Institute Inc., Cary, NC) to visualize expression patterns of pigment associated genes and transcription factors using Wards distance measure and z-score normalization of RPKM values.

### *Drosophila* wing gene regulatory network

For the candidate gene approach, genes from the *Drosophila* wing gene regulatory network were identified following a BLASTX search against the *Drosophila* peptide database. To examine the temporal expression patterns of these genes across all development stages, a two-way ANOVA was conducted in JMP, using transformed RPKM values. To normalize gene expression levels we included the *glutamate receptor* as a covariate (i.e., as an internal control). The glutamate receptor was identified as an internal control by filtering the transcriptome data for transcripts with consistent expression levels across all developmental stages (FDR *p* > 0.2).

### Comparison of butterfly transcriptomes

We identified homologous sequences from *Vanessa cardui* in other available butterfly wing transcriptomes including *Heliconius melpomene maletti*, *Heliconius melpomene cythera* (InsectBase) [32], *Junonia coenia* (datadryad.org) [38] and also the genome of *Danaus plexippus* (MonarchBase) [39]*.* The transcriptome comparison was conducted by first obtaining unigenes for the *V. cardui* transcriptome using CD-Hit suite [40] with similarity set to 0.95. CD-Hit clustered all sequences with similarity ≥95 % and retained only the longest transcript thereby removing splice variants and reducing redundancy. Each butterfly transcriptome/peptide database was compared to *V. cardui* unigenes using TBLASTX and BLASTX (for peptides in *D. plexippus*) (1E-10^-5^). Venny 2.0.2 (bioinfogp.cnb.csic.es/tools/venny/) was used to visualize results and identify transcripts common to all butterflies. We also searched for homologs of *V. cardui* unigenes in the genome of the silk moth *Bombyx mori* (Silkdb.org) [41] (BLASTX) and also the brain transcriptome from *Bicyclus anynana* [42] (TBLASTX) using the same parameters.

### Quantitative PCR validation

An independent experiment was designed to validate the transcriptome results. Wing discs and pupal wings were dissected at the same developmental stages as the transcriptome study with seven biological replicates per stage. RNA isolation was performed as described above. RNA quality was checked for degradation on a formaldehyde-agarose gel. A qPCR was also performed on the RNA with primers for the *glutamate receptor* to confirm absence of any genomic DNA contamination. cDNA synthesis was performed with an iScript kit (BioRad) in a single run for all samples using 1 μg of input RNA (20 μl reaction). An aliquot of cDNA was diluted to the equivalent of 2 ng total RNA input/μl for qPCR. Primers were designed for the following genes: *wg, sal, en, dll, ddc, pale, ebony, tan, vermillion, kf, and cinnabar* (Additional file 1: Table S1). We used cDNA (2 ng/μl) from wing samples to amplify PCR products using Accuzyme™ 2x reaction mix (Bioline). *Glutamate receptor* was selected as a housekeeping gene based on results from whole transcriptome data. The PCR products were checked for a single band (75 bp) on a 1 % agarose gel, purified using a Thermo Scientific purification kit and quantified using Nanodrop. Standard curves were generated using an initial concentration of 2 picograms of PCR product and serial 10-fold dilutions [43]. qPCR was performed using 2 μl of cDNA template with Evagreen Supermix (BIO-RAD) (10 μl reaction/well), and run on a CFX384 Real time system (Bio-rad C1000 Thermocycler) with the following conditions 95 °C 30s, 95 °C 5 s, 60 °C 5 s for 40 cycles. A bivariate regression analysis was performed in JMP to compare expression patterns for the RNA-seq and qPCR data for all 12 genes listed in Additional file 1: Table S1.

### Availability of data and materials

Raw reads used to assemble the transcriptome are deposited in the short read archive at NCBI under Bioproject accession PRJNA284000. Larval libraries used for expression analyses are listed under accession numbers SRX1603967 and SRX1603981. Pupal libraries used for expression analyses are listed under accession numbers SRX1605764 SRX1605766, SRX1605767, SRX1605768, SRX1605769 and SRX1605770.

The annotated transcriptome is available at InsectBase, www.insect-genome.com/query.php?accession=IBVcarT00001, accession number IBVcarT00001.

### Ethics statement

This research did not require any permits to obtain the butterflies and does not involve any endangered or protected species.

## Results

The final transcriptome (Table 1) comprised 89,069 contigs with a mean length of 779.8 bp and N50 of 1, 266 bp after removal of short sequences <200 bp. Mapping of the raw reads back to the transcriptome revealed that 91 % of the reads mapped to the final assembly. When larval and pupal libraries were mapped separately, 94 % of reads from the larval libraries and 87 % of reads from the pupal libraries mapped to the assembled transcriptome. For purposes of annotation, this dataset was filtered for sequences ≥ 500 bp producing 18, 491 contigs. This list was further reduced to 15, 836 unigenes using CD-Hit. The longest sequence was 15, 506 bp and the shortest was 500 bp. Average contig length was 1, 372 bp.

**Table 1:** Summary of de-novo transcriptome assembly

Assembly details	Summary statistics
Total size of transcriptome	31,689,449 bp
Total number of reads	446, 282,529
Mean no. reads for early 4th larval libraries (EL)	25,699,084
Mean no. reads for late 4th larval libraries (LL)	27,632,760
Mean no. reads for 2 day pupal libraries (EP)	23,187,027
Mean no. reads for 5 day pupal libraries (PO)	28,375,390
Mean no. reads for 8 day pupal libraries (LM)	34,585,348
Total number of contigs	89, 065
Number of contigs >200 bp	40, 638
^a^Mean contig length	779.8 bp
^a^Median contig length	446 bp
^a^Max contig length	15, 506 bp
^a^N50	1, 266 bp
Number of contigs ≥ 500 bp	18, 491
Number of unigenes	15, 836

^a^after removal of short contigs <200 bp

Of the 89, 069 contigs, a handful of contigs were identified that exhibited constant levels of expression across all developmental stages. Following BLAST searches, we identified one of these contigs as a putative *glutamate receptor*. The remaining contigs that exhibited constant expression did not match any known sequences on NCBI and are likely non-coding RNA. Quantitative PCR confirmed that expression of the *glutamate receptor* did not vary across developmental stages (*p* > 0.05). This gene was used as a covariate for subsequent qPCR analyses.

### Correlation of qPCR and RNA-seq data

**Fig. 2:**
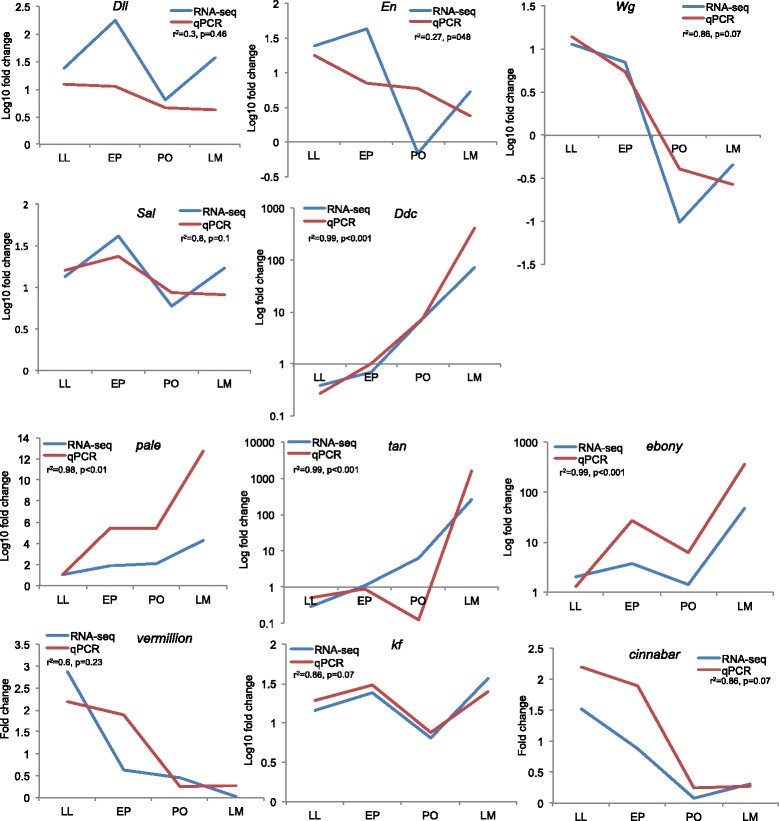
RNA-Seq and qPCR data showing fold change expression for patterning and pigment genes. Fold change is calculated for individual genes at each developmental stage relative to early 4^th^ instar. Correlation coefficient and *p* value for the hypothesis r = 0 are also presented

A bivariate analysis of fold change in expression relative to the early 4^th^ larval stage for all twelve genes (including the *glutamate receptor*) revealed that the RNA-seq and qPCR results are largely consistent with each other (Additional file 2: Figure S1). Examination of fold change for each gene individually reveals very similar expression patterns and a high correlation between the RNA-seq and qPCR analysis for most genes (Fig. 2). Weaker correlations were found for genes expressed at very low levels (e.g. *dll* and *en*). One of the pigment genes, *Vermillion* exhibited opposite patterns of expression between the qPCR and RNA-seq results.

### Top differentially expressed transcripts

**Fig. 3:**
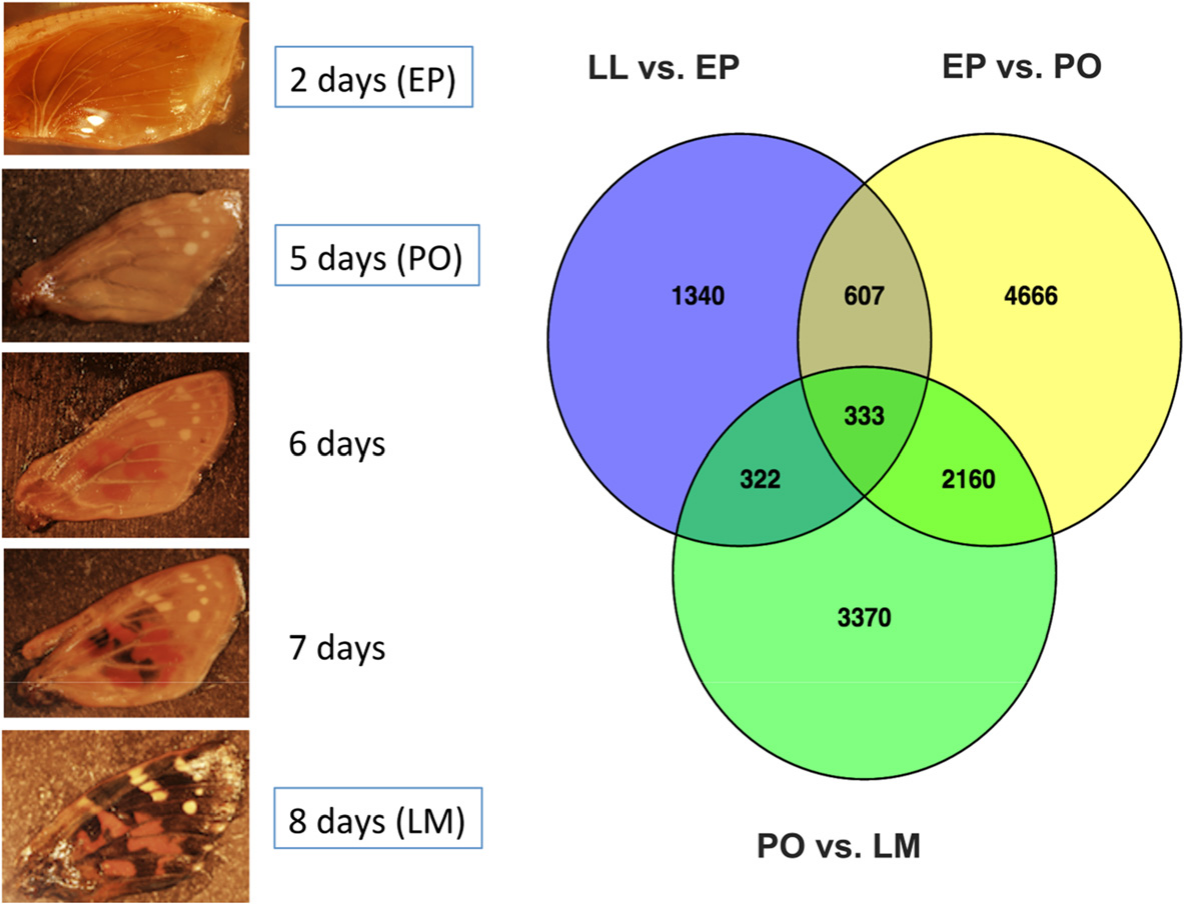
Venn diagram depicting the abundance of differentially expressed transcripts (FDR *p* < 0.001) for each comparison between wing developmental stages. Images illustrate various stages of wing development (in days post-pupation) for *Vanessa cardui*. Sampled stages for the transcriptome analysis are highlighted in blue boxes

**Fig. 4:**
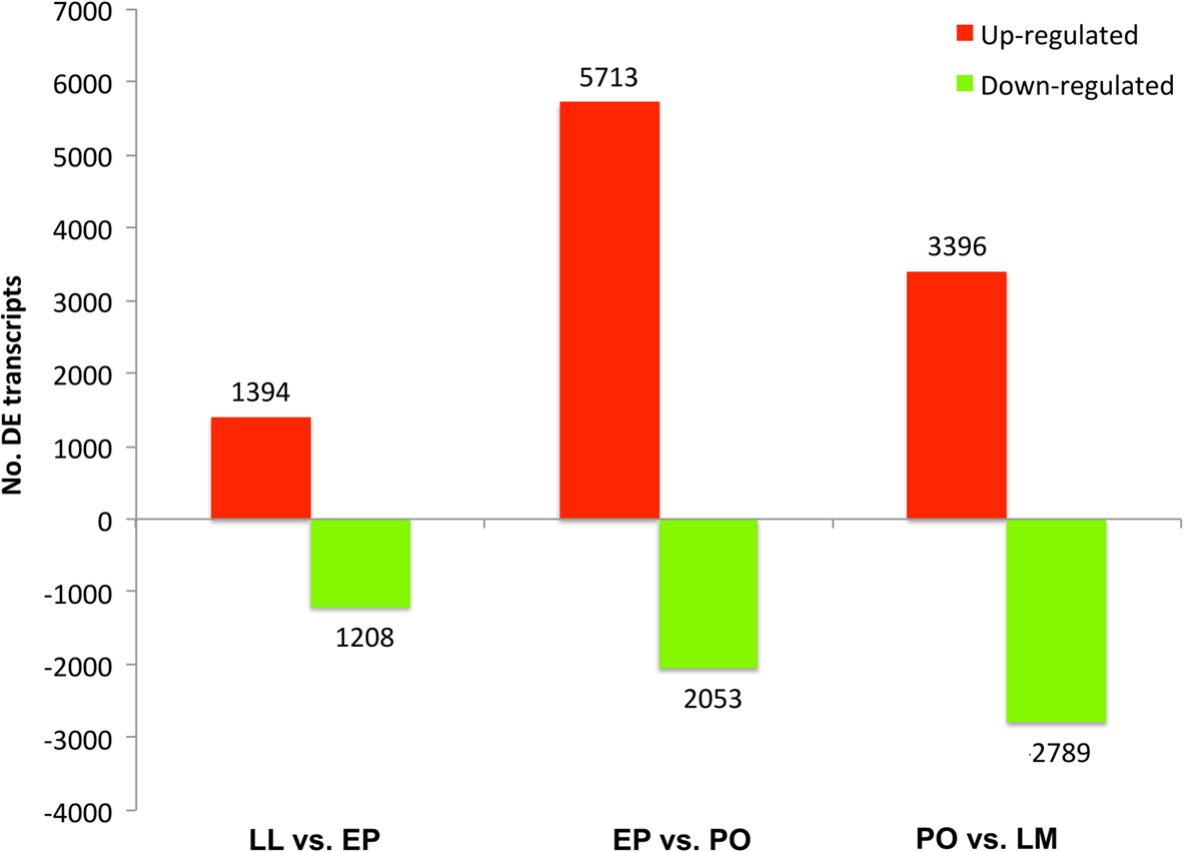
Abundance of differentially expressed transcripts (FDR *p* < 0.001) between wing developmental stages

Using an FDR cut off *p* < 0.001, we identified a total of 2, 602 transcripts differentially expressed between LL vs. EP stages. After filtering for transcripts ≥ 500 bp, this list was reduced to 1, 260 transcripts, of which 1, 065 were annotated with genes names and 832 were annotated with GO terms. The comparison between EP vs. PO stages revealed that 7, 766 transcripts were differentially expressed. Following filtering for size, this list was reduced to 2, 397 transcripts, of which 1, 852 were annotated and 1, 419 received GO terms. For the PO to LM transition a total of 6, 185 transcripts were differentially expressed. Filtering transcript length ≥ 500 bp reduced this list to 1, 582 transcripts, of which 1, 199 were annotated and 926 received GO terms. The Venn diagram (Fig. 3) illustrates the number of transcripts found in common between the developmental stages. Overall, fewer transcripts were found in common between the LL to EP transition than between the other developmental stages. Furthermore, development of the wing from EP to PO stages produced the highest number of differentially up-regulated transcripts, while the number of transcripts significantly down-regulated increased during wing development (Fig. 4).

Genes showing the highest fold change between LL vs. EP included genes from the Osis family, cuticular and ecdysone induced proteins. Transcripts most strongly down-regulated included aldehyde oxidases*,* larval serum and cuticle proteins. From EP to PO stages, the aldehyde oxidases were significantly up-regulated as were a number of cuticular proteins and chitinases. One of the most strongly down-regulated genes at this stage was *E75* (Ecdysone inducible protein 75). During the PO to LM transition, the ammonium transporter (*Amt*) exhibited the largest fold change increase and several aldehyde oxidases were strongly down-regulated. For all stages there were many uncharacterized transcripts that were significantly up or down regulated. These transcripts exhibited the largest fold change for both the EP vs. PO and PO vs. LM stages (Additional file 3: Table S2 and Additional file 4: Table S3 lists all DE genes and GO annotations respectively).

### Drosophila wing gene regulatory network

We next examined whether any of the genes from the *Drosophila* wing GRN were included in the top differentially expressed transcript list (FDR *p* < 0.001). Both *en* and *hh* were significantly down-regulated from the larval to early pupal stage. *Hedgehog* and *wg* were also significantly down regulated during the transition from the early pupal to pre-ommochrome stage. There was no evidence of differentially expressed genes during the pre-ommochrome to late melanin stages. Other genes known to be important for wing development in *Drosophila* and potentially wing patterning in butterflies were also significantly down-regulated, including *Notch, Wnt6* and *Wnt10* (Additional file 3: Table S2). Though not differentially expressed, *antennepedia* and *aristalless* were observed in the transcriptome of *V. cardui*. We were unable to identify *optix*, a gene strongly associated with red pigmentation in *Heliconius* butterflies [27, 44].

**Fig. 5:**
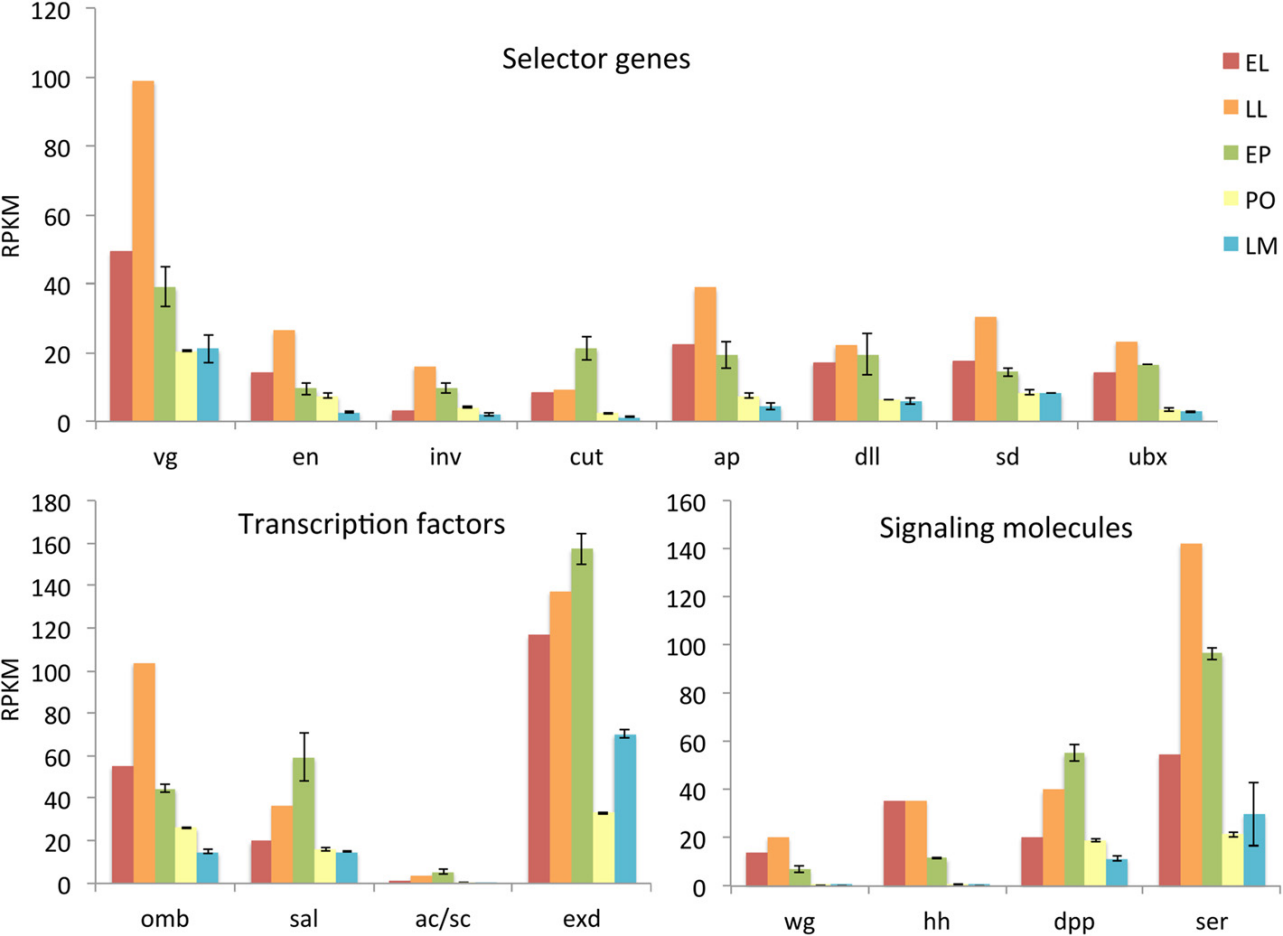
RNA-Seq expression patterns for the different functional groups of the wing GRN. Larval stages (EL and LL) each represent one pooled biological replicate (5 individuals); pupal stages (EP, PO and LM) represent two biological replicates of 3-4 pooled individuals. Error bars represent 1 SD from the mean

Though only a few genes from the *Drosophila* wing GRN were identified as differentially expressed at a stringent transcriptome-wide FDR, all genes with the exception of *abd-A* were present in the wing transcriptome. We examined the temporal expression patterns of these genes during wing development. For most patterning genes, peak expression occurred during the late larval and early pupal stages (Fig. 5). Relative to the glutamate receptor, expression of all patterning genes declined significantly (gene x development stage; *p* < 0.001) with the exception of *extracdenticle* (*exd*)*,* which was up-regulated during the late melanin stage.

### Pigment genes differentially expressed during wing development

**Fig. 6:**
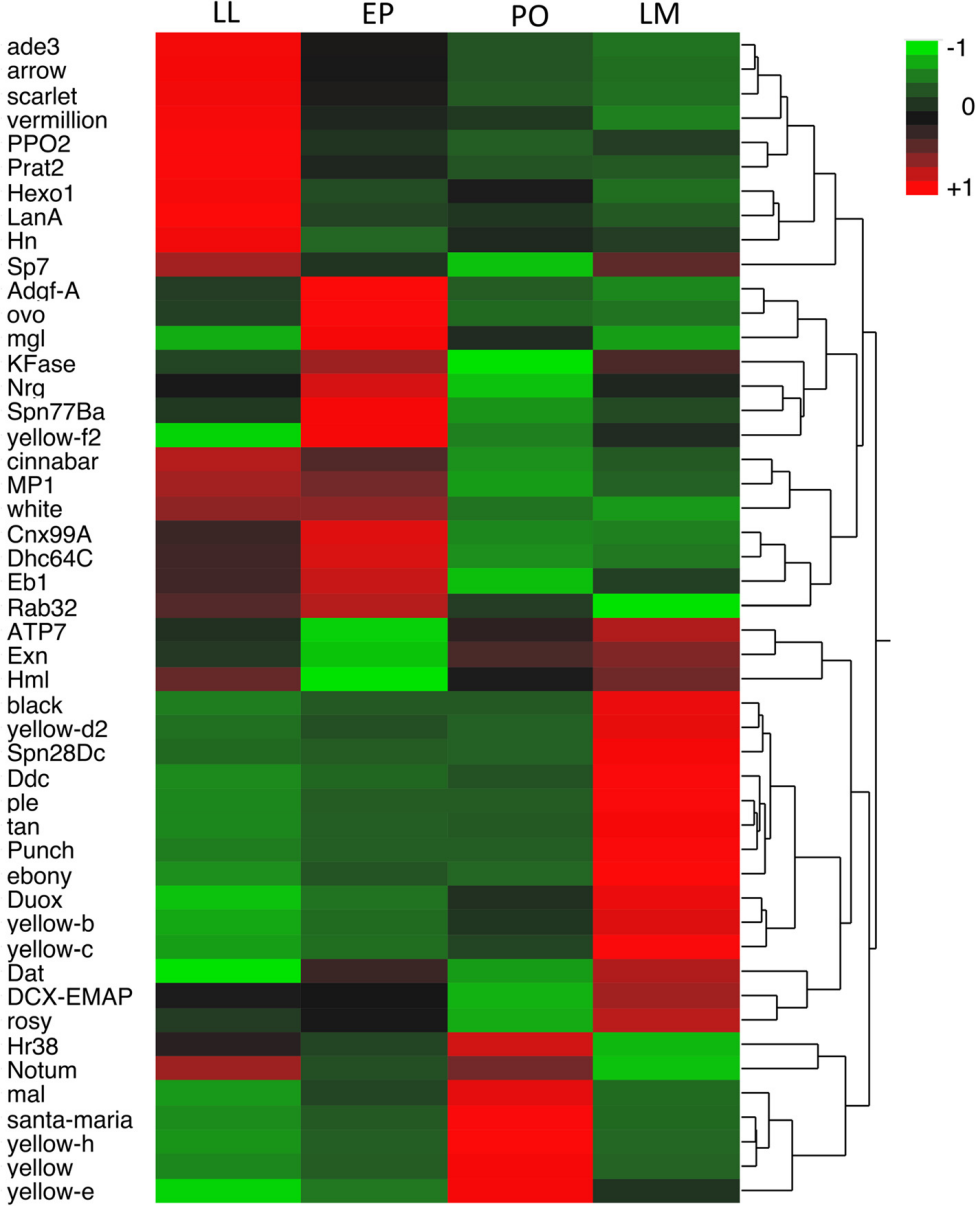
Heatmap of genes associated with pigmentation expressed during wing development. Each gene is differentially expressed (FDR *p* < 0.001) between at least two developmental stages (See Additional file 5 Table S4 for full details)

To understand the process of wing color patterning in *V. cardui*, we focused our analysis on genes involved in pigmentation, and also transcription factors that may regulate expression of pigment-associated genes. In total, we identified 130 pigment genes of which 50 were differentially expressed (FDR *p* < 0.001) (Fig. 6). Not surprisingly, a larger proportion of pigment-associated genes were up regulated compared to those down regulated during wing development. During the transition from larval to early pupal stages, genes involved in melanin biosynthesis were significantly up-regulated; *yellow-f2* exhibited the largest fold change increase in expression (Fig. 6). Other melanin genes (e.g. *Ddc*) were significantly down-regulated, along with genes involved in the pteridine (*Henna, Prat2, adenosine3*), and ommochrome (*vermillion* and *scarlet*) pathways. We also identified several pigment granule genes (*claret, garnet, ruby*, *dor*); however, only *dor* was differentially expressed (Additional file 5: Table S4). During the transition from EP to PO stages, the *yellow* genes exhibited the largest fold change increase. Melanin immune response genes (*Nrg, Spn77Ba*) were among the most strongly down-regulated in addition to genes involved in the ommochrome pathway (*cinnabar, scarlet* and *white*). A number of genes from the pteridine pathway were significantly up-regulated during this stage (*rosy, mal, henna*). During the late melanin stage, most of the genes strongly up-regulated were those involved in the melanin pathway including: *black, yellow-d2* and *pale.* Genes involved in the ommochrome (*Kfase)* and pteridine pathway (*rosy*) were also strongly up-regulated, although the ommochrome gene *vermillion* and the melanin gene *yellow-y* were significantly down-regulated (see Fig. 6 and Additional file 5: Table S4 for full details).

### Transcription factors differentially expressed during wing development

**Fig. 7:**
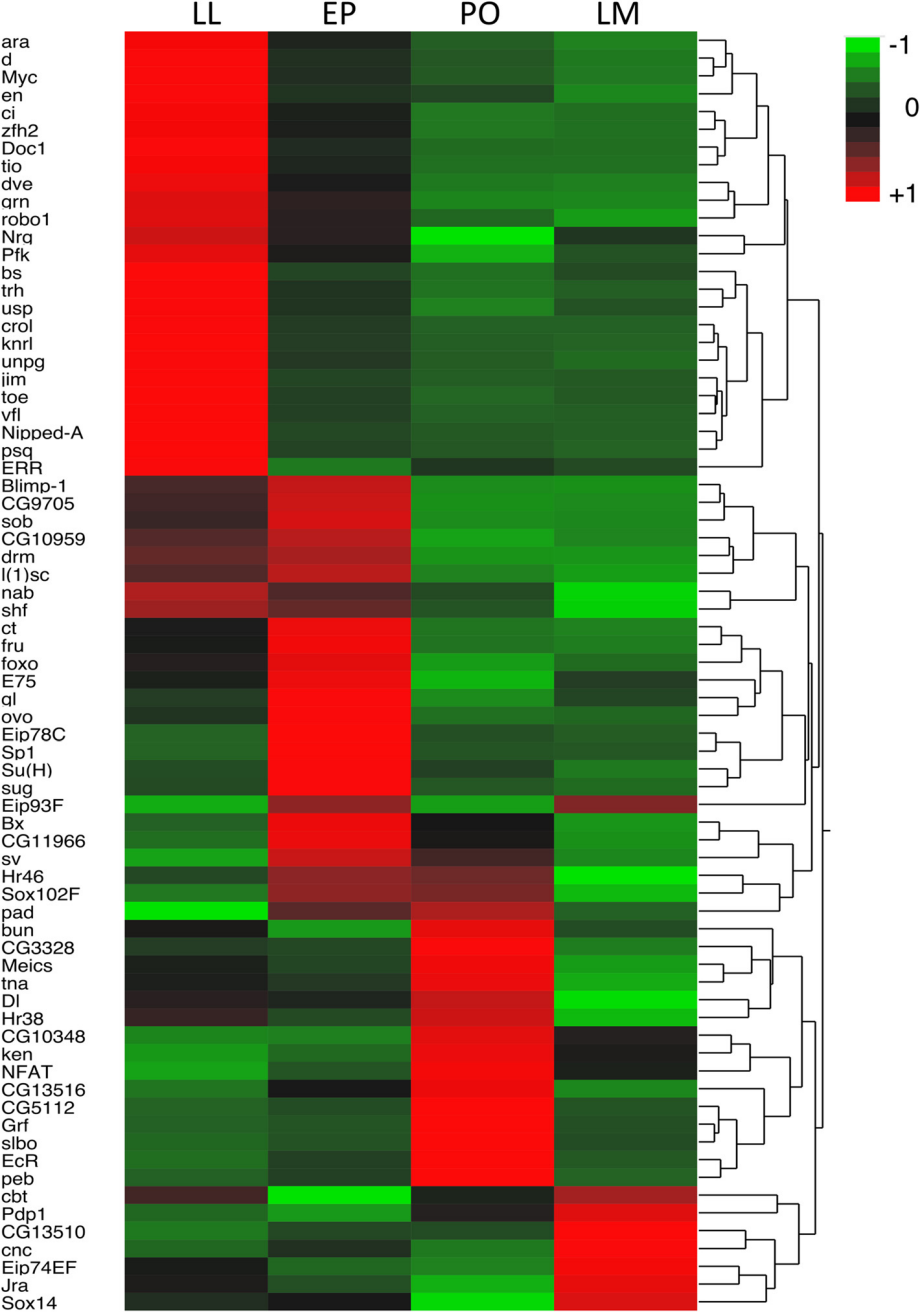
Heatmap of transcription factors expressed during wing development. Each gene is differentially expressed (FDR *p* < 0.001) between at least two developmental stages (See Additional file 6 Table S5 for full details)

Overall we identified 248 transcription factors, of which 72 were differentially expressed (FDR *p* < 0.001) (Fig. 7). In contrast to pigment genes, a higher proportion of transcription factors were significantly down-regulated versus up-regulated during wing development (Additional file 6: Table S5). Many of these transcription factors are known to play important roles in hormonal signaling. Ecdysone receptor and a number of ecdysone-induced proteins were identified among the top differentially expressed transcription factors, showing dynamic expression patterns during wing development. Ecdysone-induced proteins that were significantly up-regulated during the LL to EP stages (e.g. *Eip93F*) were significantly down-regulated from the EP to PO stages and then up-regulated again during the transition to the late melanin stage. Ultraspiracle, which forms a dimer with Ecdysone receptor (EcR), was significantly down-regulated during the LL to EP stages prior to the up-regulation of EcR during the PO stage. EcR was subsequently down-regulated during the final stages of wing development and pigmentation. *Shaven* and *Sp1* also exhibited dynamic expression patterns; both were strongly up-regulated during the LL to EP stage but significantly down-regulated from PO to LM stages. Overall, the largest proportion of transcription factors was up-regulated during the transition from EP to PO stages. Interestingly, many of the up-regulated transcription factors during the pre-ommochrome stage are known to interact with Notch signaling including *slow border cells, bunched, Delta* [45] *and pebbled/hindsight* [46]*.*

### Comparison of butterfly transcriptomes

**Fig. 8:**
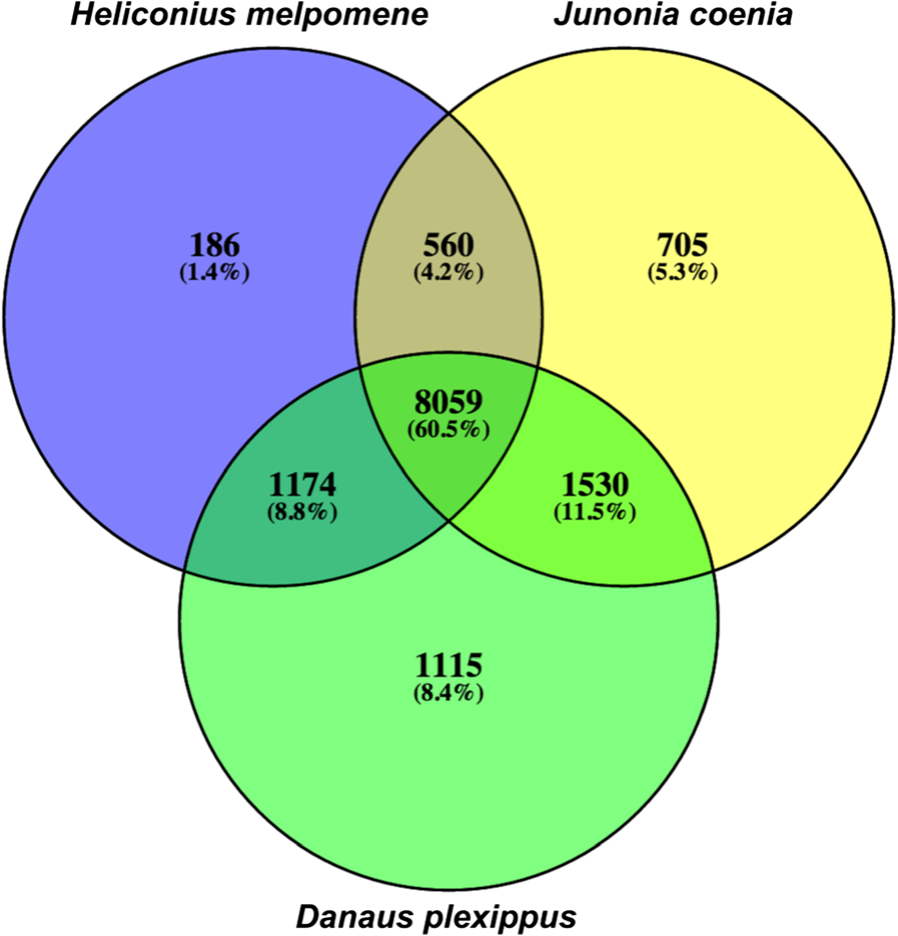
Venn diagram depicting the abundance of transcripts in common with *Vanessa cardui* (1E-10^-5^) for three species of butterfly (*Heliconius melpomene, Junonia coenia* and *Danaus plexippus*)

Finally, we performed a comparative analysis to examine conservation of wing development genes between different butterfly species. We conducted a TBLASTX search of *V. cardui* transcriptome (15, 836 unigenes) against the wing transcriptomes of *Heliconius melpomene* (72, 234 contigs)*, Junonia coenia* (16, 251 contigs) and a BLASTX search against the peptide database of *Danaus plexippus* (15, 130 contigs). In total, 9, 979 transcripts from *H. melpomene* (*cythera*: 6, 227, *maletti*: 3, 752), 10, 854 from *J. coenia*, and 11, 878 from *D. plexippus* produced significant hits to *V. cardui* unigenes. In *Bombyx mori* we identified 11, 291 contigs with significant sequence similarity to *V. cardui*. The three butterfly species shared 8, 059 unigenes from *V. cardui* (Fig. 8). Of these 8, 059 unigenes, 7, 001, had significant hits to flybase and 2, 081 were among the top differentially expressed transcripts during wing development in *V. cardui* (710 during LL vs. EP, 1, 299 during EP vs. PO and 863 during PO vs. LM). Overall we identified 28 contigs common to all four butterflies that received no significant hits to NCBI even when using more permissive parameters (1E-3). Six of these contigs were among the top differentially expressed transcripts in *V. cardui* (Additional file 7: Table S6). These 28 contigs were also identified in the brain transcriptome of *Bicyclus anynana* indicating these specific transcripts are expressed in different tissues. Interestingly, just 12 of these contigs were identified in the genome of *Bombyx mori*, and only 2 were among the top differentially expressed transcripts compared to all 6 identified in brain tissue from *B. anynana*. Surprisingly, we identified 12, 632 unigenes from *V. cardui* in the brain transcriptome of *B. anynana*. Furthermore, 812 (74 %) of the non-annotated transcripts differentially expressed in *V. cardui* were not found in the brain, suggesting these novel transcripts may be unique to wings.

## Discussion

Here, we describe the first transcriptome analysis of wing development in the painted lady butterfly, *Vanessa cardui* and examine expression dynamics of genes involved in tissue patterning, pigmentation and gene regulation. One goal of this study was to compare expression patterns of genes from the wing GRN in *Drosophila* with genes involved in pigmentation. Many genes from the *Drosophila* wing GRN are expressed in butterfly wing patterns suggesting these developmental genes may function as a pre-patterning template for genes involved in pigmentation [9, 25]. The temporal expression patterns of these genes have not been examined in butterfly wings thus it remained unclear how their expression corresponds with the timing of pigment genes. If patterning genes are involved in regulating pigmentation, their expression may be significantly up-regulated at some stage during wing color pattern development. For example, expression of *spalt* and *distal-less* are associated with melanin pigmentation [28, 47]; therefore, upregulation of these genes may coincide with increased expression of melanin genes.

Contrary to our expectation, we did not observe these wing-patterning genes significantly up-regulated at any stage during pupation. In fact, many of these genes were significantly down-regulated during pupal development. Expression of these patterning genes generally peaks during larval and early pupal stages, subsequently declining during wing development. Any potential role these genes play in regulating pigmentation likely occurs during these earlier developmental stages, long before pigmentation becomes visible on the wing. Our results also revealed that genes characterized in the *Drosophila* wing GRN (with the exception of *abd-A*) are also expressed in the wings of *V. cardui*. Many of these genes have been identified in the wings of ants and pea aphids [10, 11] and more recently *Bombyx mori* [48]. Whether this wing GRN is functionally conserved in these insects is currently unknown.

### Largest number of transcripts upregulated during the EP to PO transition

To further explore genes involved in wing development we examined the top differentially expressed transcripts between late larval and early pupal stages (LL vs EP), early pupal to pre-ommochrome (EP vs. PO) and pre-ommochrome to late melanin stages (PO vs LM). We found that the highest number of up-regulated transcripts occurred during the EP to PO stage indicating that this is a dynamic period during wing development. At this stage, white scale patches are clearly visible on the wing and ommochrome pigments are deposited shortly after. The transition from LL to EP stages was marked by a larger turnover of transcripts compared to the other pupal stages, which shared a greater proportion of genes in common. This is likely due to significant morphological changes occurring between the larval and pupal wings. As reported in *B. mori*, we also observed an increasing number of transcripts down-regulated during the progression of wing development [48]. Therefore the process of wing maturation appears to be largely regulated by gene silencing.

### Temporal dynamics of pigment gene expression

As expected, an increasing number of pigment-associated genes were up-regulated during wing development. We predicted that if patterning genes regulate pigment genes, we should observe temporal co-expression of these genes during wing color pattern development. As patterning genes peak in expression during larval and early pupal stages, expression of some pigment genes should also be up-regulated at this time. Surprisingly, genes involved in the melanin pathway (*yellow-f2, yellow, yellow-h, ple*) were significantly up-regulated during the LL to EP transition, almost a week before melanin pigments become visible on the wing. However, not all melanin genes were up-regulated; the enzyme Dopa-decarboxylase (*Ddc)* was significantly down-regulated along with genes associated with the melanin immune response [49]. Melanin pigment is produced when tyrosine is converted by pale (tyrosine hydroxylase) to dopa and further to dopamine by Ddc [50]. Polyphenol oxidases (PO) and enzymes from the yellow gene family are thought function downstream to convert dopa and/or dopamine to black pigment. There are contrasting views regarding whether black melanin is derived primarily from dopa or dopamine, although recent RNAi experiments of melanin genes indicate that dopamine is a necessary precursor for black pigmentation [51]. Our results suggest that the enzyme specifically required for melanin pigmentation is repressed during early pupation. Interestingly, *Dat,* the enzyme responsible for producing colorless cuticle was significantly up-regulated [50] along with Megalin (*mgl*) a multi-ligand receptor which regulates cuticle integrity and localization of yellow [52]. Loss of *Dat in B. mori* and *mgl* in *Drosophila* results in ectopic melanin pigmentation, highlighting the critical role of these genes in restricting melanization [52, 53]. Taken together, these results suggest that partial up-regulation of the melanin pathway is required for pre-patterning wing regions fated for melanization. Alternatively, these genes could be involved in other developmental processes during early pupation such as cuticle development [54–56].

We also examined whether any homologs of pigment granule genes were significantly up-regulated in addition to the up-regulation of pigment genes. In *Drosophila* these genes are involved in the biogenesis of pigment granules which house either the brown ommochromes or red drosopterins [57, 58]. Although we identified several putative pigment granule genes (*claret, garnet, ruby, dor*) in the wing transcriptome, only deep orange (*dor*) was significantly up-regulated (during early pupation). The other granule genes were not differentially expressed at any developmental stage. It still remains unclear whether the model proposed for eye pigmentation in *Drosophila* is consistent with the process of scale pigmentation, as pigment granules have not been identified in butterflies [25, 27].

During the pre-ommochrome stage prior to red pigmentation, many genes involved in ommochrome synthesis (brown, yellow, red pigments) were strongly down-regulated including *cinnabar, scarlet, white* and *kfase*. These results support earlier studies indicating that expression of most genes in the ommochrome pathway are up-regulated during larval wing development and decline during pupation [25]. For both ommochrome and melanin genes, there is a significant time lag between the up-regulation of pigment genes and onset of pigment synthesis in the wings. This lag suggests that other genes may also be involved in regulating pigmentation. Examination of spatial patterns of gene expression in pupal wings might resolve this apparent discrepancy in timing between peak transcript levels and pigmentation. Another possibility is that pigmentation enzymes are regulated post-translationally [25].

We observed significant up-regulation of pteridine enzymes involved in *Drosophila* red eye pigmentation, xanthine dehydrogenase (*rosy*)*,* maroon-like, (*mal*)*,* and phenylalanine hydroxylase (*henna*) coincident with down-regulation of ommochrome genes [59, 60]. Pteridines are well described in the Pieridae, with different pterins responsible for white, yellow and orange pigments due to variation in absorption of violet and UV light [61, 62]. Pterins are also cofactors for ommochrome biosynthesis in pigment cells of ommatidia in *Drosophila* as well as enzymes involved in growth and differentiation [63]. Whether the pteridine pathway serves any functional role during wing pigmentation in nymphalid butterflies is currently unknown. A recent study revealed that *rosy* is up-regulated in the red morph of *Junonia coenia* implicating involvement of both pteridine and ommochrome pathways in the development of red pigmentation [38].

Transition from the early pupa to pre-ommochrome stage was also characterized by significant up-regulation of all the major genes involved in the melanin pathway including several members of the *yellow* family. Although expression of genes from the melanin pathway is strongly associated with black pigmentation, some of these genes are also expressed in red and yellow wing tissues in *Heliconius* and *Papilio* butterflies [27, 64]. Up-regulation of melanin and pteridines during the pre-ommochrome stage may indicate involvement of these pathways in non-melanic pattern elements in *V. cardui*.

As expected, most genes significantly up-regulated during the late melanin stage were those from the melanin pathway, although the most strongly up-regulated gene was *black* which functions in suppressing melanin synthesis [65, 66]. Increased expression of *black* along with *Dat* and *ebony* may prevent melanization in non-black regions of the wing as shown in *Heliconius*, where *ebony* and *Dat1* are up-regulated in red and yellow tissues respectively [27]. We also observed strong up-regulation of *yellow-d2,* which increased almost 200-fold in expression. In *Heliconius*, y*ellow-d* is up-regulated in red tissues, revealing that not all yellow genes are associated with black pigmentation [27]. Whether *yellow-d* and *yellow-d2* share similar functions in pigmentation is currently unknown. The function of *yellow* genes in pigmentation is poorly understood as the same paralog can exhibit contrasting functions in different species; for example, *yellow-d* is associated with melaninized tissue in *Bombyx mori* [67] and unmelanized tissue in *Heliconius* species [27, 68]. Interestingly, in *V. cardui*, yellow genes strongly down-regulated during the pre-ommochrome stage (*yellow-f2* and *yellow-d2*) were significantly up-regulated during the late melanin stage, while the opposite trend was observed for *yellow-y*, *yellow-e* and *yellow-h*. Collectively, these results suggest different functional groups of *yellow* genes, which may play either activating or repressive roles in melanin pigmentation.

### Transcription factors differentially expressed during wing development

To explore candidate genes that may potentially regulate scale color fate we identified differentially expressed transcription factors during wing color pattern development. During early pupation and scale determination [69], we observed significant up-regulation of *shaven/sparkling* and *polis au dos* (*pad*). In *Drosophila*, *shaven/sparkling* represent mutations in two distinct enhancers of *D-Pax2* that not only influence eye morphology (*sparkling*), but also control development of sensory bristles of the pupal retina (*shaven*) [70]. *Pad* is also involved in bristle development and appears to function as a negative regulator of *achaete-scute* [71]. It has been proposed that bristles and scale cells are homologous structures based on shared expression of *achaete-scute* [72]. Although *achaete-scute* was not differentially expressed during early pupation, up-regulation of bristle development genes supports the assertion that these structures are homologous.

*Ovo/shavenbaby*, a gene involved in oogenesis, epidermal differentiation and trichome formation [73], was also among the most significantly up-regulated transcription factors. In *Heliconius* butterflies, *ovo* is differentially expressed in hindwings, indicating a possible role of this gene in color patterning [27]. This appears to be the case in *Drosophila* where *ovo* regulates expression of *yellow* during the development of pigmented denticles [73]. Interestingly, we also observed significant up-regulation of *yellow-y* during the early pupal stage. Whether *ovo* is also involved in regulating expression of *yellow* or other pigment genes in butterfly wings is currently unknown.

During the pre-ommochrome stage, we found that many up-regulated transcription factors have generalized roles in cell migration, growth and differentiation (*slow border cells (slbo), pebbled/hindsight (peb), bunched (bun)* and *delta (Dl*)). The specific function of these genes in the context of butterfly wing development has not been investigated; therefore, we can only speculate on their potential roles. Interestingly, several of these genes interact with the signaling molecule Notch. The Notch receptor has been implicated as an important regulatory molecule for specifying butterfly wing patterns [74, 75], although the specific signaling pathway leading to pattern development is still unknown. It has been proposed that *slbo/bun/Notch* may function as a conserved signaling cassette in regulating cell fate boundaries. In *Drosophila*, this signaling cassette specifies anterior/posterior identity of follicle cells during oogenesis [45, 76]. *Bun* also functions in restricting Notch activity to the wing margin, producing a notched wing phenotype when mutated [76]. Our finding that *bun, slbo and dl* were significantly up-regulated during the PO stage suggests this signaling cassette may also be conserved in Lepidoptera, however we did not observe differential expression of Notch. We did identify eight putative splice variants of Notch. Several of these were significantly down-regulated during the other pupal stages. If these genes are part of a conserved Notch signaling cassette, then activation during the pre-ommochrome stage may specify positional information of pattern elements just prior to visibility on the wing (approximately 24 h later).

### Transcription factors involved in hormonal regulation

Hormones play a fundamental role regulating insect metamorphosis and can also influence pigmentation of butterfly wing pattern elements [38, 77, 78]. Ecdysone signaling begins with the release of ecdysone (20E). Ecdysone (20E) is a ligand for the ecdysone receptor and a heterodimer of EcR (Ecdysone Receptor) and Usp (Ultraspiracle) [79]. Hormone regulation of gene expression occurs when the heterodimer binds ecdysone response elements in the promoters of ecdysone-responsive target genes [80]. In *V. cardui,* we observed contrasting patterns of expression for EcR and ecdysone-responsive genes (*E75C, Eip93F, Eip74EF, Eip78C*). EcR expression peaked during the pre-ommochrome stage and declined during the late melanin stage; however, the opposite pattern was observed for ecdysone-responsive genes. Previous work demonstrates that EcR can have a repressive function when ecdysone levels are reduced [81]. Thus, ecdysone levels may decline during the pre-ommochrome stage, resulting in EcR repression of these target genes. Studies on pigmentation in lepidopteran larvae suggest that expression of melanin and ommochrome genes are regulated by ecdysone-induced transcription factors [79, 82, 83]. Work in *Drosophila* also shows that *Ddc* contains an ecdysone response element (EcRE) that binds EcR [84]. Other ecdysone inducible genes, like *E75,* have been proposed as potential regulators of *Ddc* during larval cuticle development in *Manduca sexta* [79]. There are comparatively few studies examining ecdysone regulation of pigment genes during pupal development, therefore the potential role of these transcription factors is largely unknown. Further investigation is required to identify whether other pigment genes also possess EcRE’s, and if ecdysone-inducible transcription factors regulate their expression during wing color pattern development.

## Conclusions

We have assembled the first wing transcriptome for *Vanessa cardui* and identified a suite of genes involved in patterning, pigmentation and gene regulation, including many genes not previously described in butterflies. Some of these genes include transcription factors, which were significantly up-regulated during wing development. These factors may be involved in regulating wing color pattern development. In addition to ommochrome and melanin genes, we identified genes from the pteridine pathway, indicating that nymphalids may utilize this pathway for generating pigments. Our analysis also identified genes from the *Drosophila* wing GRN; genes from this network show similar temporal expression dynamics to enzymes involved in the ommochrome pathway which peak early during wing development. Although some melanin genes were also up-regulated during this developmental stage, *Ddc*, which is required for melanin pigmentation was significantly down-regulated. These results suggest that the melanin pathway is repressed during early pupation; however, up-regulation of some melanin genes indicates functionality in aspects other than pigmentation. Finally, our comparative analysis of transcriptomes across butterfly species identified a common set of transcripts with no known homologs to other animals and may represent novel genes unique to Lepidoptera.

## Additional files

Additional file 1: Table S1. Primers used for qPCR validation. (DOC 31 kb) Additional file 2: Figure S1. Bivariate analysis of fold change expression for RNA-Seq and qPCR. Fold change is relative to the early 4^th^ larval wing for all developmental stages across all genes. The regression shows a strong correlation for results obtained using these two different methods. Data points for RNA-Seq are the result of one pooled (5 individuals) biological replicate for larval stages and two pooled (3-5 individuals) biological replicates for pupal stages. Data points for qPCR are based on 7 biological replicates. Correlation coefficient, *p* value for the hypothesis r = 0, and sample size for gene expression data for 12 genes are also presented (*dll*, *en*, *wg*, *sal, vermillion*, *kf, cinnabar*, pale, *ddc*, *ebony*, *tan* and *glutamate receptor*). (PDF 719 kb) Additional file 3: Table S2. Differentially expressed (DE) transcripts (≥500 bp) for each comparison between wing development stages (LL vs. EP, EP vs. PO, PO vs. LM). Information provided on fold change, FDR *p*-value, RPKM and gene name for each contig. Up-regulated genes are highlighted in pink and down-regulated genes are highlighted in green. This table also includes the complete list of all 9, 376 differentially expressed transcripts. (XLSX 645 kb) Additional file 4: Table S3. BLAST2GO PRO gene ontology classification for differentially expressed genes (FDR *p* < 0.001) for each comparison between wing developmental stages (LL vs. EP, EP vs. PO, PO vs. LM). (XLSX 751 kb) Additional file 5: Table S4. Differentially expressed genes (FDR *p* < 0.05) identified as pigment genes from AMIGO2 for each comparison between wing developmental stages (LL vs. EP, EP vs. PO, PO vs. LM). Information provided on fold change, FDR *p*-value, RPKM and gene name for each contig. Up-regulated genes are highlighted in pink and down-regulated genes are highlighted in green. (XLSX 84 kb) Additional file 6: Table S5. Differentially expressed genes (FDR *p* < 0.05) identified as transcription factors from the Animal Transcription Factor Database for each comparison between wing developmental stages (LL vs. EP, EP vs. PO, PO vs. LM). Information provided on fold change, FDR *p*-value, RPKM and gene name for each contig. Up-regulated genes are highlighted in pink and down-regulated genes are highlighted in green. (XLSX 42 kb) Additional file 7: Table S6. Comparison of butterfly transcriptomes reveals 28 contigs common to *Vanessa cardui, Junonia coenia, Danaus plexippus and Heliconius melpomene* which received no significant hits to NCBI (1E-3). Contigs highlighted in grey are differentially expressed in *V. cardui* across the developmental stages, shown in red (up-regulated) or green (down-regulated). Blast results for all comparisons to *V. cardui* including *Bicyclus anynana* brain transcriptome and *Bombyx mori* are also presented. (XLSX 4847 kb)
