# Supplementary material for: Transcriptome analysis of the painted lady butterfly, Vanessa cardui during wing color pattern development
Source: BMC Genomics. 2016 Mar 31;17:270. doi: 10.1186/s12864-016-2586-5 (PMC4815134; doi:10.1186/s12864-016-2586-5)
Supplement: Additional file 1: Table S1. — Primers used for qPCR validation. (DOC 31 kb) [file 12864_2016_2586_MOESM1_ESM.doc]

**Additional file Table S1**. Primers used for qPCR validation.

| **Gene** | **Primer sequences** |
| --- | --- |
| ***Glutamate receptor*** | Forward -TGGTATCGTCGCCATATTCG  Reverse - GGAGAATATCAGCGCACCGA |
| ***Wingless*** | Forward - AAAAGCTGGCGAACCAAACA  Reverse - GGTTGCGTGAACTCCTGGAT |
| ***Spalt*** | Forward - GAAAACGATGGAGGGCAAGA  Reverse - AGTCCATGCTGCAGTCGTCA |
| ***Engrailed*** | Forward - GTACACCTGCACCACCATCG  Reverse - CGGTGAGTTCGGTTGGACTTT |
| ***Distal-less*** | Forward - GGCTTGGGATGTAAAGGTTGG  Reverse - TGGTGGCTTCACGTCACAA |
| ***Ddc*** | Forward - ACGACATCGAGCGCGTTATA  Reverse - GCTGTCGGGAAATAGGCGT |
| ***Tan*** | Forward - ATCCCCACGCAAGAAGACAG  Reverse - GCAAGTGACCCGCATAGCA |
| ***Pale*** | Forward - CTCGTAGATGACGCCCGCT  Reverse - GTGCACGAGCCTCTTCAAGC |
| ***Ebony*** | Forward - CATCCTGACTTTGGCCGTCT  Reverse - TGCCAGCGAACAAGATGAGA |
| ***Kf*** | Forward - GCATGTGGTCGACGAGGTTT  Reverse - TCGCTTGCTGTGGTAACGAA |
| ***Vermillion*** | Forward - AATGCGTGAACCCAACGAAG  Reverse - GGATCGGTATATTTCCCGCC |
| ***Cinnabar*** | Forward - ACGAGGACATCGAGTGTCCC  Reverse – AATGGAACGCCCTCGTACAT |
