# Supplementary figures and images for: Transcriptome analysis of the painted lady butterfly, Vanessa cardui during wing color pattern development
Source: BMC Genomics. 2016 Mar 31;17:270. doi: 10.1186/s12864-016-2586-5 (PMC4815134; doi:10.1186/s12864-016-2586-5)

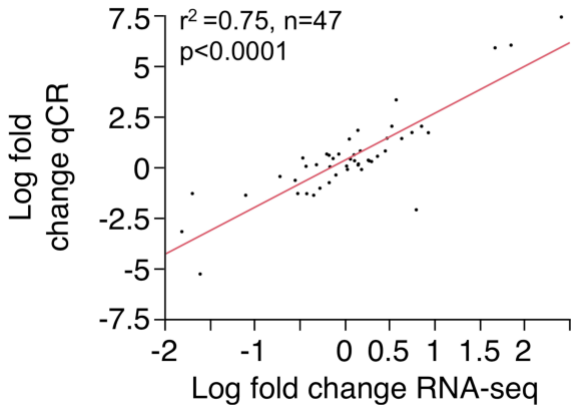

Supplement: Additional file 2: Figure S1. — Bivariate analysis of fold change expression for RNA-Seq and qPCR. Fold change is relative to the early 4th larval wing for all developmental stages across all genes. The regression shows a strong correlation for results obtained using these two different methods. Data points for RNA-Seq are the result of one pooled (5 individuals) biological replicate for larval stages and two pooled (3-5 individuals) biological replicates for pupal stages. Data points for qPCR are based on 7 biological replicates. Correlation coefficient, p value for the hypothesis r = 0, and sample size for gene expression data for 12 genes are also presented (dll, en, wg, sal, vermillion, kf, cinnabar, pale, ddc, ebony, tan and glutamate receptor). (PDF 719 kb) [file 12864_2016_2586_MOESM2_ESM.pdf]
